# Epidemiological tracing of bovine tuberculosis in Switzerland, multilocus variable number of tandem repeat analysis of *Mycobacterium bovis* and *Mycobacterium caprae*

**DOI:** 10.1371/journal.pone.0172474

**Published:** 2017-02-21

**Authors:** Giovanni Ghielmetti, Simone Scherrer, Ute Friedel, Daniel Frei, Dominique Suter, Lukas Perler, Max M. Wittenbrink

**Affiliations:** 1 Institute of Veterinary Bacteriology, Vetsuisse Faculty, University of Zurich, Switzerland; 2 Federal Food Safety and Veterinary Office, Bern, Switzerland; St Petersburg Pasteur Institute, RUSSIAN FEDERATION

## Abstract

**Background:**

After 15 years of absence, in 2013 bovine tuberculosis (bTB), caused by *Mycobacterium* (*M*.) *bovis* and *M*. *caprae*, reemerged in the Swiss dairy cattle population. In order to identify the sources of infection as well as the spread of the agents, molecular-epidemiologic tracing by MIRU-VNTR analysis in combination with spoligotyping was performed. A total of 17 *M*. *bovis* and 7 *M*. *caprae* isolates were cultured from tuberculous bovine lymph nodes and analyzed with a set of 49 genetic markers by using automated capillary electrophoresis.

**Results:**

The outbreak in the western part of Switzerland was caused by *M*. *bovis* spoligotype SB0120. With the exception of four single-locus variations observed in MIRU 20, the MIRU-VNTR profiles of the 17 *M*. *bovis* isolates were identical, indicating a single source of infection. *M*. *bovis* detected in one archival bovine specimen from the outbreak region showed an identical MIRU-VNTR profile, suggesting persistence of the agent in a dairy herd for nearly fifteen years. The outbreak in the eastern part of Switzerland was caused by *M*. *caprae* spoligotype SB0418. All Swiss *M*. *caprae* isolates showed the Lechtal-type MIRU-VNTR profile, described as endemic in wild ruminants and in dairy cattle in Austrian bordering regions. This suggests the agent was most likely introduced by Swiss dairy cattle summering on Austrian pastures.

**Conclusions:**

The present study is the first MIRU-VNTR analysis of Swiss bTB mycobacterial isolates. The genotyping assay was found to be highly discriminating and suitable for the epidemiological tracing of further outbreaks. These findings will contribute to the development of an international MIRU-VNTR database aiming to improve bTB surveillance.

## Introduction

Bovine tuberculosis (bTB) is an important zoonosis with serious implications for livestock farming and public health. In Europe, the most common causative agent of bTB is *Mycobacterium (M*.*) bovis* and to a lesser extent, *M*. *caprae* [1–3]. Despite considerable efforts over decades to eradicate bTB by intensive test and slaughter programs, it remains enzootic and—beyond that—in some European regions it is actually considered a serious re-emerging disease [3–5]. Switzerland has been faced with bTB for many years. A nationwide disease control program was first attempted in 1896. The Swiss government made tuberculin tests accessible to the cantonal veterinary offices and provided financial support for voluntary farm testing [6]. In the 1930’s, the Swiss Federal Council decided to increase this approach to tuberculosis control with the aim of eradicating the disease from farm animals [7]. This proved to be an onerous process due to the high prevalence in some dairy herds of up to 50%, and the dearth of information about the status of the wild animal population [8]. After more than a decade of different cantonal bTB control approaches with the aim of minimizing the economic losses, in March 1950 a federal law for the eradication of bTB was approved. This intervention entailed the culling of all tuberculin skin reactor animals. Nine years later, in December 1959, Switzerland was officially declared free from bTB according to the requirements of section 3.2.3.10 of the OIE Animal Health Code [9]. Following this, the requirements for disease surveillance involved meat inspection at abattoir level combined with comparative intradermal tuberculin tests on imported livestock. In the past 50 years, only sporadic within-one-herd outbreaks of bTB were registered. In March 2013, however, an outbreak involving nine dairy farms in the western region of Switzerland was detected. The causative agent was identified as *M*. *bovis*. Spoligotyping and multi-locus variable number tandem repeat analysis (MLVA) targeting mycobacterial interspersed repetitive units (MIRUs) and other variable-number tandem repeats (VNTRs) of the cultured isolates were performed.

In November 2013, a new outbreak in the eastern region of the country was detected. The causative agent was *M*. *caprae* and all isolates showed the Lechtal-type MIRU-VNTR profile, described as endemic in wild ruminants and in dairy cattle in Austrian bordering regions [10]. This agent was therefore most likely introduced by Swiss dairy cattle summering on Austrian pastures. In order to identify the infection source and correlation between affected animals, it is fundamental to investigate genetic differentiation of the pathogens. To date, many molecular typing methods have been described for *Mycobacterium tuberculosis* Complex (MTBC) pathogens. Spoligotyping (spacer oligotyping) is a widely used PCR-based method that enables simultaneous detection and typing by targeting the highly polymorphic direct repeat (DR) region in the MTBC genome. In fact, even when more elaborate analysis targets, such as single nucleotide polymorphism (SNPs) or large sequence polymorphisms are available, spoligotyping remains an essential tool in veterinary mycobacteriology. The low cost, digitalization of results which can be easily shared among laboratories and the creation of an international database (www.Mbovis.org.) are the main reasons for the importance of spoligotyping [11]. This method shows a remarkable weakness in the differentiation of some large strain families, however. A more discriminative and accurate genotyping technique for MTBC isolates is MLVA targeting polymorphic MIRU-VNTRs. MLVA has recently emerged in veterinary mycobacteriology because of its robustness and greater resolution compared to spoligotyping, enabling the identification of individual mycobacterial strains and, consequently, the identification of transmission dynamics among livestock or between wild and domestic animals in a cost-effective way. Multiple sets of informative VNTR markers were described for *M*. *tuberculosis* [12–16] and also for *M*. *bovis* [17–19]. Many of these have shown a high discriminatory power but their application remains restricted to mycobacterial samples from a given geographical area. Many authors have consequently emphasized the necessity of defining discriminatory panels of gene *loci* for different mycobacterial species, which are known to exhibit genetical variability in different geographical regions [17, 19–22]. The purpose of the present study was to genotype *M*. *bovis* and *M*. *caprae* strains isolated from two recent outbreaks of bovine tuberculosis in Switzerland using the spoligotyping technique combined with a panel of 49 MIRU-VNTR markers. Additionally, this study aimed to establish a high-throughput automated procedure enabling epidemiological tracing of tuberculous mycobacteria from cattle.

## Materials and methods

### Ethics statement

Based on Swiss federal regulations (TSV 916.401, VSFK 817.190 and VHyS 817.190.1) bTB is an eradicable disease under mandatory surveillance and monitoring. Suspicious samples are submitted at the National Reference Laboratory for confirmation of bTB diagnosis by bacteriological culture and PCR. Analysis of animal specimens was therefore carried out within an official context, meaning that no animals were killed for the purposes of this research project and ethical approval was not necessary. The privacy rights of farmers were fully protected and the obtained data were de-identified.

### Specimen collection

From March 2013 until November 2014, approximately 9800 animals in the western part of Switzerland and 3900 animals in the eastern Cantons from epidemiologically linked farms were tested for bTB. The *M*. *bovis* outbreak was monitored exclusively using tuberculin test while for the second outbreak, caused by *M*. *caprae*, the Interferon–gamma Assay (Bovigam, Thermo Fischer Scientific, Reinach, Switzerland) was used in addition to skin testing for defined contact premises. In total, lymph nodes from 534 cattle from 203 dairy herds suspected of bTB were submitted for mycobacteriological analysis. 248 out of 534 animals were IDTB or IGRA positive. Of the remaining 286, 84 were doubtful reactors at IDTB and for 202 animals no data records were available. Forty-eight cows showed visible lesions compatible with tuberculosis, 304 did not show visible lesions and for the remaining animals no data were available. An average of three lymph nodes was processed from each animal. Lymph nodes were of retropharyngeal, mandibular, bronchial or mediastinal origin. Gross organ lesions including caseous abscesses of the lung or liver and granulomatous lesions of the pleural and peritoneal serous membranes were also examined.

### Culture and identification of mycobacteria

Two grams of each tissue specimen were homogenized in 20 ml NaCl (0.9%) by using a rotating-blade macerator system (T18 Digital Ultra-Turrax IKA, Staufen, Germany) and centrifuged 15 min at 3000 x *g*. After discarding the supernatant, the sediment was decontaminated with slight modifications to the method described by De Kantor and co-workers [23]. Briefly, the sediment was thoroughly resuspended in 4.0 ml H_2_SO_4_ (4%), incubated 15 min at ambient temperature, and neutralized by adding 5.65 ml NaOH (1 M). Afterwards, 20 ml phosphate buffered saline solution (PBS, pH 7.4) was added and the suspension centrifuged 15 min at 3000 x *g*. The final sediment was resuspended in 2.5 ml PBS and used as inoculum. 40 μl of inoculum was suspended in 360 μl ATL buffer (Qiagen, Hilden, Germany) and transferred onto a bead beating matrix in a 2 ml microtube (Omni International, Kennesaw, USA). Genomic DNA extraction was performed as described in the section titled “mycobacterial DNA”. Purified DNA was used for direct MTBC detection by *artus M*. *tuberculosis* PCR Kit using 7500 Fast real-time PCR system (7500 Fast; Applied Biosystems, Zug, Switzerland) according to the manufacturer’s protocol (Qiagen). Finally, 0.25 mL of decontaminated inoculum was transferred to a BBL Löwenstein-Jensen supplemented with PACT (Polymixin B, Amphotericin B, Carbenicillin, Trimethoprim) and a BBL Stonebrink agar slants (Becton, Dickinson & Company, Allschwil, Switzerland). Cultures were incubated at 37°C for eight weeks. In addition, two BBL MGIT liquid media tubes supplemented with Bactec MGIT 960 growth supplement, BBL MGIT PANTA (Polymyxin B, Amphotericin B, Nalidixic acid, Trimethoprim, Azlocillin) antibiotic mixture (BD) and 50 μg/ml f.c. sodium-pyruvate were inoculated with 0.5 ml each and incubated up to seven weeks in a BACTEC MGIT 320 incubator (BD) [24]. Cultures showing growth of suspicious mycobacterial colonies were checked for acid-fast bacilli after Ziehl-Neelsen staining. Pure mycobacterial cultures were identified as MTBC by *artus M*. *tuberculosis* PCR Kit (Qiagen). Species identification was performed by reverse line blotting using the GenoType MTBC kit (HainLifescience, Nehren, Germany) according to the manufacturer’s instructions. *M*. *tuberculosis* H37Rv, *M*. *bovis* BCG Tice ATCC 27289 and *M*. *bovis* BCG Pasteur ATCC 35734 were used as controls.

### Mycobacterial DNA

Mycobacteria were harvested from 1.5 ml MGIT cultures by centrifugation for 10 min at 13000 x *g*. The sediment was suspended in 180 μl ATL buffer (Qiagen), transferred onto a bead beating matrix in a 2 ml microtube (Omni International, Kennesaw, USA), heated 30 min at 99°C and subjected to a mechanical lysis consisting of four cycles of 45 seconds (s) at 6,500 rpm with a Precellys 24 homogenizer (Bertin Technologies, Montigny, France). After adding 20 μl of Proteinase K (Qiagen) the mixture was incubated 12 h at 56°C and 10 min at 95°C under constant shaking. Automated DNA preparation from these samples was performed on the QIAcube instrument in accordance with the QIAamp cador Pathogen Mini Kit protocol (Qiagen). DNA concentration in the final eluate was measured by reading the absorbance at 260 nm using a NanoDrop 2000c Spectrophotometer (Thermo Fisher Scientific), diluted to a concentration of 100 pg/μl and stored at -20°C until use.

### Spoligotyping and MLVA

Spoligotyping was performed by using a commercial microarray system with integrated data analysis (Alere Technologies, Jena, Germany) [25]. Amplification of the DR region of MTBC was performed on a Veriti Thermal Cycler (Applied Biosystems). Each reaction contained 0.5 U of *Taq* Polymerase S, 1 x amplification buffer with supplemental 1.5 mM MgCl_2_ (Genaxxon BioScience, Ulm, Germany), 0.1 mM of each dNTP, 1 μM of the 5'-biotinylated primers DRa/DRb and 2 ng purified mycobacterial DNA in a final volume of 10 μl. The PCR started with an initial 3 min polymerase activation step at 94°C followed by 30 cycles of 94°C for 20 s, 55°C for 20 s, and 72°C 30 s, and a final extension step of 72°C for 5 min. DNA-DNA Hybridization on ArrayStrips (Alere Technologies) was performed following the manufacturer’s instructions. Reading of the microarray strips and automatic processing of the obtained data were subsequently run by an ArrayMate reader, using the Iconoclust version 4.4 and the ArraySoftware 16047 (Alere Technologies). The latter includes normalization to the background level, automatic spot recognition, and signal intensity output in a grey value median table. Signal intensities higher than 0.3 (on a scale from 0 to 1.0) were considered positive for the respective probe. The signals for all 43 probes were reduced to a binary code, with “1” for positive and “0” for negative [25]. The spoligotype patterns were assigned according to the international nomenclature [11]. *M*. *tuberculosis* H37Rv, *M*. *bovis* BCG Tice ATCC 27289 and *M*. *bovis* BCG Pasteur ATCC 35734 were used as reference strains.

The MLVA analysis was performed on a total of 49 MTBC gene *loci* showing variable numbers of tandem repeat units. In addition to the standard 24 markers panel used for *M*. *tuberculosis* typing according to Supply *et al*. [20], 25 MIRU-VNTR markers were identified through a literature search. Six of them, VNTR 3336 [26], VNTR 1451 [27], VNTR 1612 [28], VNTR 0024, VNTR 3663 and VNTR 2990 [16], were subsequently excluded because of unsatisfactory PCR amplification. Finally, a set of six *loci* were designed by the authors using the Tandem Repeats Finder web site (S1 and S2 Files). The latter described *loci* were named following the VNTR nomenclature convention proposed by Smittipat *et al*. [14], based on the first four of seven digits of the nucleotide number in the genome of the H37Rv strain (S1 Dataset).

The single markers were amplified by simple PCR. Each reaction mixture contained 1 x HotStar *Taq* Master Mix Kit, 1x Q-Solution (Qiagen), 0.5 μM of each primer pair and 200 pg purified mycobacterial DNA in a final volume of 10 μl. The PCR started with a 15 min polymerase activation step at 95°C followed by 40 cycles of 94°C for 60 s, 59°C for 60 s, 72°C for 60 s, and a final extension step of 72°C for 10 min. 10 μl of each PCR amplification products were analyzed using a capillary electrophoresis device (QIAxcel, Qiagen), using the OH1700 AM10sec method with a QX DNA high-resolution cartridge, QX 15 bp– 3 kb alignment marker and QX 100bp– 2.5 kb size marker. Peak size assignment and allele code exportation was performed with the QIAxcel ScreenGel Software version 1.3.0 (Qiagen).

### Modification of the QIAxcel System for high-throughput analysis

As previously described [29, 30], the QIAxcel advanced System is highly accurate in sizing PCR products up to 600 bp. Overestimation by sizing larger fragments was observed, however, in particular for *loci* 3820, 4155, 3232 and 2163a. The expected allelic code obtained by sequencing could therefore not be confirmed by the QIAxcel analysis. To minimize the size of the amplicons new primer pairs were placed closer to the repeat units (primer sequences shown in S1 Dataset). These changes solved the discrepancy problems in two cases, namely in *locus* 3820 and *locus* 4155, where the size of the fragment corresponding to allele 0 was shortened from 273 to 139 bp and from 644 to 113 bp respectively, without consequences to the PCR reproducibility.

**Fig 1:**
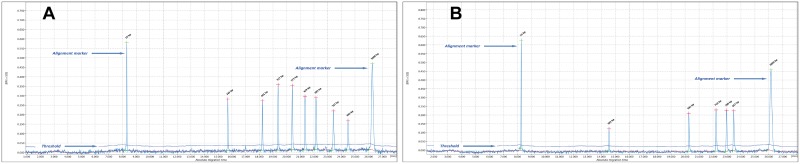
Allelic ladder specific for *locus* VNTR 3232 (A) and VNTR 2163a (B). The commercial size marker provided by the manufacturer was replaced with band ladders generated by different repeat numbers of *locus* VNTR 3232 (A) and VNTR 2163a (B). Allelic ladders represent a satisfactory solution for the problem of overestimation error due to the different nucleotide sequences between the size marker and analyzed fragment.

However, for fragments larger than 600 bp (*locus* 3232 and *locus* 2163a) overestimation and the consequent allele shift of the tested isolates persisted. For this reason a size marker, or allelic ladder, composed of amplicons of known size with different repeat unit numbers was developed specifically for each of the *loci* described above (Fig 1).

### Confirmation of results accuracy in case of double bands

The fragments were separated by conventional gel electrophoresis in a 1% pulsed field certified agarose (Bio-Rad Laboratories) stained with UltraPure ethidium bromide (Thermo Fischer Scientific) and visualized by UV light. For DNA sequencing, PCR products were purified (QIAquick PCR Purification Kit, Qiagen GmbH, Hilden, Germany) and subsequently sequenced by Microsynth AG (Balgach, Switzerland).

## Results

**Fig 2:**
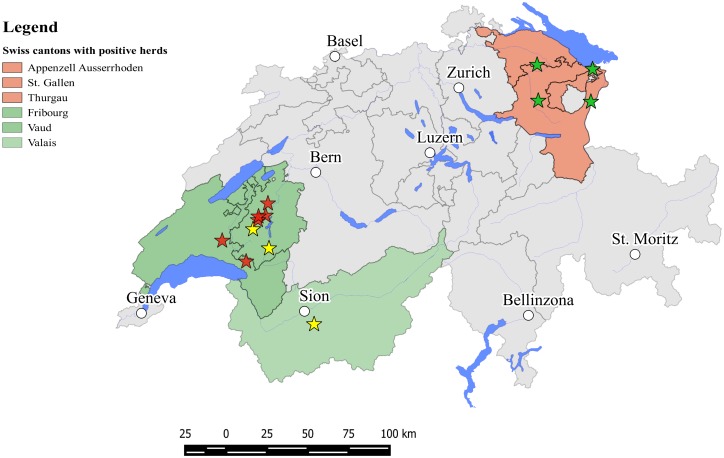
Map of Switzerland showing the geographical origin of the positive samples. Green stars indicate *M*. *caprae*, red stars *M*. *bovis*, both positive by RT-PCR testing and successively in culture; yellow stars indicate samples positive by direct RT-PCR though mycobacteria were not isolated in culture. Swiss Cantons where cases of bTB were detected are highlighted. The free software QGIS was used for map design (Source of layers: Swiss Federal Office of Topography).

Twenty-four culture-confirmed bTB cases isolated from March 2013 to November 2014 were analyzed by reverse line blotting, spoligotyping and MLVA. *M*. *bovis* and *M*. *caprae* were isolated in 17 respectively in 7 lymph node pools out of 534 samples on MGIT. Three isolates, two *M*. *caprae* and one *M*. *bovis*, grown on liquid culture could not be isolated on BBL Stonebrink agar slants. Typical growth of acid-fast bacilli could be observed on Löwenstein-Jensen media for only thirteen of the twenty-four isolates. *M*. *bovis* isolates were collected from six farms, all of them located in the western part of Switzerland (Canton Fribourg: 4 farms, 10 isolates; Canton Vaud: 2 farms, 7 isolates) and *M*. *caprae* isolates originated from four farms located in three Cantons (Appenzell Ausserrhoden 1 farm, 4 isolates, St. Gallen 2 farms, 2 isolates and Thurgau 1 farm, 1 isolate) (Fig 2).

Mycobacteria from five MTBC RT-PCR positive samples could not be isolated in culture and therefore further analysis by spoligotyping and MLVA was not performed due to the low amount of available mycobacterial genomic DNA. Among these samples, three were originating from three contact farms where culture confirmed animals could not be detected (Fig 2). Epidemiological contact tracing analysis, based on the Swiss national stock movement database, confirmed the origin of the pathogen and linked the three farms to the outbreak caused by *M*. *bovis* SB0120.

### Spoligotyping and MLVA

Spoligotype analysis revealed a SB0120 and SB0418 profile for all the *M*. *bovis* and *M*. *caprae* isolates tested respectively. Spoligotype SB0120 is characterized by the absence of spacers 3, 9, 16, and 39–43; SB0418 by the lack of spacers 1, 3–16, 28, and 39–43.

For the panel of seventeen *M*. *bovis*, seven *M*. *caprae* and three MTBC reference strains, a PCR product was obtained for the complete set of tested MIRU-VNTR *loci*. Therefore, typability was 100% for the 49 chosen markers. Two clusters of profiles (one for *M*. *bovis*, one for *M*. *caprae*) were detected for the Swiss isolates among the analyzed *loci* (Table 1). In both cases single-locus variation (SLV) could be found in four *M*. *bovis* isolates and in one *M*. *caprae* isolate.

**Table 1:** Allele profiles of Swiss *M*. *bovis* and *M*. *caprae* isolates compared with three reference strains in the 24 MIRU-VNTR standard panel (A) and in the 25 MIRU-VNTR additional *loci* (B).

**A**																									
		**154**	**424**	**577**	**580**	**802**	**960**	**1644**	**1955**	**2059**	**2163b**	**2165**	**2347**	**2401**	**2461**	**2531**	**2687**	**2996**	**3007**	**3171**	**3192**	**3690**	**4052**	**4156**	**4348**
*M*. *tuberculosis* H37Rv		2	2	4	3'	1	3	2	2	2	5	3	4	2	3	6	1	3	3	3	3	5	5	2	2
*M*. *bovis BCG Pasteur* ATCC 35734		2	0	6	2'	2	2	3	1	2	3	5	2	2	5	4	2	5	3	3	3	2	5	0	2
Swiss field strain *M*. *bovis*		2	0	5	3	2	2	3	1	3	3	4	3	4	7	4	2	5	1	3	3	2	5	1	2
Swiss field strain *M*. *caprae*		2	4	5	2	2	6	4	2	2	5	5	3	4	3	4	2	4	3	2	3	1	3	3	2
**B**																									
	**79**	**569**	**917**	**1121**	**1281c**	**1305**	**1443**	**1895**	**1907**	**1982a**	**1982**	**2074**	**2163a**	**2372**	**2705**	**3155**	**3189**	**3232**	**3239**	**3291**	**3351**	**3594**	**3820g**	**4120**	**4155g**
*M*. *tuberculosis* H37Rv	6	2	2	4	2	2	1	4	2	2	5	3	2	2	1	3	2	3	2	1	2	3	3	2	2
*M*. *bovis BCG Pasteur* ATCC 35734	10	1	2	3	2	1	2	4	1	3	3	2	10	1	2	3	2	5	1	4	2	3	7	2	11
Swiss field strain *M*. *bovis*	8	1	2	3	2	1	2	4	1	4	3	2	10	1	2	3	2	6	1	4	2	3	11	2	7
Swiss field strain *M*. *caprae*	3	0	1	3	2	1	2	4	1	5	3	1	8	1	1	3	2	11	1	2	2	1	7	2	5

Nomenclature in accordance with the European Union Reference Laboratory for Bovine Tuberculosis, VISAVET Health Surveillance Centre, Complutense University of Madrid (EURL).

**Fig 3:**
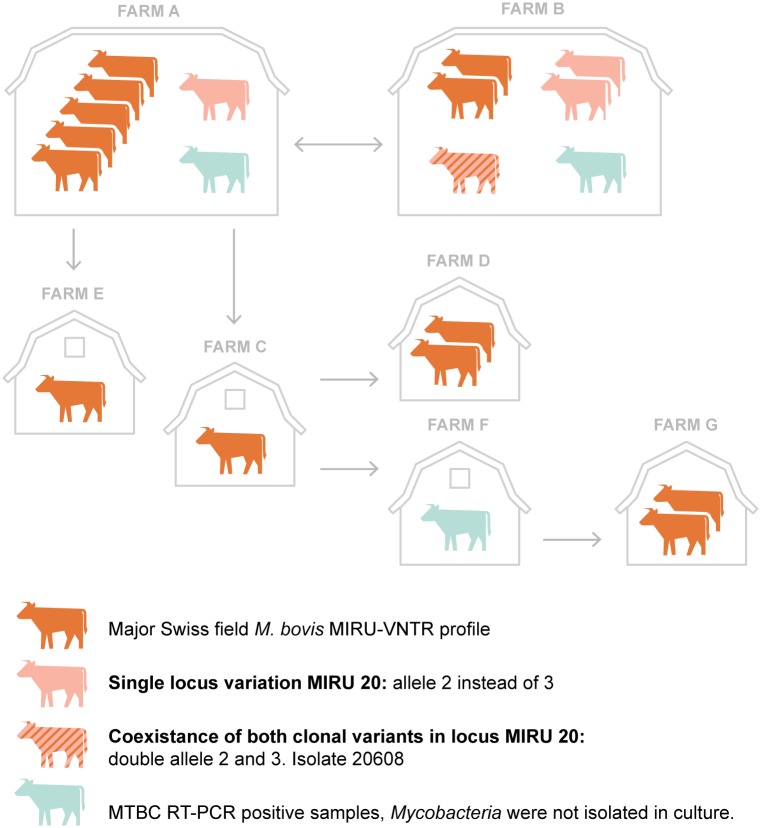
Probable bTB transmission by which the infection spread between cattle of different farms in the outbreak caused by *M*. *bovis* SB0120. Arrows indicate movements of confirmed infected animals. Since cattle from two farms (only RT-PCR positive samples) spent the summer months on pasture together with animals originating from both farm A and farm B, unequivocal epidemiological contact tracing at single animal level was not demonstrable. These two premises are therefore not displayed.

Thirteen *M*.*bovis* isolates shared an identical MIRU-VNTR profile in all the 49 *loci* analyzed. Three isolates showed a SLV in *locus* VNTR 2059 (alias MIRU 20), whereas one isolate (No. 20608) showed a double band profile in the described *locus* (Fig 3). Sequencing of the two amplicons revealed the loss of one of the three repeat units. It is interesting to note that, during the present epidemiological investigation mycobacterial DNA from an archival bTB specimen, a cow slaughtered in 1998, was analyzed and showed an identical MIRU-VNTR profile with the *M*. *bovis* cluster from the recent outbreak. The origin of the cow slaughtered in 1998 was farm A.

All *M*. *caprae* isolates exhibited identical MIRU-VNTR profiles revealing an infection with one clonal population. A subtle genetic variation was observed in one isolate from Appenzell Ausserrhoden, displaying a double allele (allele 2 and 0) in *locus* VNTR 802 (alias MIRU 40). Sequencing of the two amplicons revealed nine base pair deletions in addition to 32 single point mutations in the flanking sequence of the smaller PCR product (S3 File).

## Discussion

The epidemiological background as well as the genotyping results of the *M*. *bovis* and *M*. *caprae* isolates provide evidence that the recent outbreaks of bTB in Switzerland are etiologically unrelated i.e. the outbreak in the western part of Switzerland was caused by *M*. *bovis* SB0120 whereas the outbreak in the eastern part of Switzerland was caused by *M*. *caprae* SB0418. We therefore describe a slight genetic heterogeneity among the *M*. *bovis* isolated, which was observed through the analysis of a large number of gene *loci*. It is noteworthy that, in a routine MLVA analysis targeting the most discriminatory eight *loci* proposed by the European Union Reference Laboratory (EURL), the genetic heterogeneity within the *M*. *bovis* isolates would not have been detected [21]. Considering, on one hand, the very low prevalence of MTBC in Swiss cattle population and on the other hand the high similarity of *M*. *bovis* MIRU-VNTR profiles, microevolution from a common ancestor strain can be assumed. We could not prove that microevolution has occurred in cow 20608, since animals from farm A and B were regularly in contact, spending the summer months on the same pasture, and the two patterns were observed in both herds (Fig 2). Single locus variation or double locus variation (DLV) is a well-known phenomenon in *M*. *tuberculosis* [13, 31] as well as in *M*. *bovis* [17, 22, 27], although the simultaneous detection of both allele variants is a rarity. In contrast to a mixed infection, defined as the simultaneous infection by two or more *M*. *bovis* strains with evident distinct DNA fingerprints, cow 20608 was infected with two bacterial subpopulations. Interestingly, variability was detected only among the standard panel of 24 *loci* and not in the commonly called hypervariable *loci* VNTR 1982, VNTR 3232, VNTR 3820 or VNTR 4120. This supports the current understanding that, depending on the regional evolution of MTBC agents, a different set of MIRU-VNTR should be selected and adopted according to the geographical area of interest. The variability observed in *M*. *bovis* for *locus* VNTR 2059 in this study, despite the small number of analyzed isolates, suggests a possible remarkable discriminatory power for the mentioned *locus*. In contrast to our results, the same marker has been defined as poorly discriminatory in many studies and is not present in the panel of eight *loci* recommended by the EURL for *M*. *bovis* strains analysis [18, 19, 21].

To date, information about micro-evolutionary events and variability of *M*. *bovis* MIRU-VNTR profiles over time in infected cattle is lacking [32, 33]. The spatiotemporal occurrence and frequency of genotype variation in animals therefore remain a vital part of the definition of genetic distance and epidemiological linkage between close related isolates. Since 1980, surveillance of bovine tuberculosis in Switzerland has been based exclusively on meat inspection. In 1998, in response to a detected cow (the archival specimen included in the present study), the premise (farm A) was screened twice with the single intradermal cervical test at an interval of 60 days. One year later, the herd was re-tested and all animals resulted negative. From 1998 to 2013 no further cases of bovine tuberculosis were identified in the region. In 2013, the index-case of the present study, an eleven year old dairy cow that never left farm A, showed visible lesions and was detected at meat inspection [34]. Animals of farm A and contact farms were tested with the tuberculin test and since the majority of the cows originating from farm A were reactors, the entire herd has been culled. Whether the *M*. *bovis* strain persisted in the region over decades or has been reintroduced in Switzerland more recently is a yet unanswered question. As there is no evidence for recent bTB infections in Swiss wildlife or among farm workers, however, persistence of *M*. *bovis* SB0120 in cattle for at least fifteen years seems to be the most reasonable epidemiological elucidation of the outbreak. Moreover, against the background of our results, the persistence of the strain among the local cattle population could explain the SLV, since the time span is reasonably long enough for the emergence of clonal variants. In order to elucidate a possible impact resulting from the genetic change occurred in *locus* MIRU 20, mapping of the mentioned *locus* in reference strain *M*. *bovis* AF2122/97 was performed. MIRU 20 is located upstream of a gene that encodes a putative flavoprotein. The present study demonstrates no evidence for advantageous metabolic changes resulting from the described micro-evolution event. In fact, the emerged clonal variant does not seem to outcompete the progenitor strain. The small number of samples analyzed impede our ability to draw conclusions about its impact on the present population.

*M*. *bovis* SB0120, also known as BCG-like, is the most frequent spoligotype observed in Italy and France, with 54% and 26% prevalence respectively [21, 35], and reported as highly prevalent in Belgium, Germany, Netherlands, and Spain. Thus, our results confirm that spoligotyping alone does not allow the epidemiologically unequivocal characterization of *M*. *bovis* strains. In contrast, by comparing the obtained MIRU-VNTR allele profile with isolates from central Europe an interesting finding could be ascertained. Limiting the comparison to the eight highly discriminatory markers proposed by the EURL [21], one clustering profile could be found in the French database, a *M*. *bovis* SB0120 strain isolated in 1989 from a cat originating from a central region in France. This MIRU-VNTR profile could not be observed in any other isolates, even by comparing other spoligotype patterns. The origin and movements of the cat could not be traced back. The majority of more than one thousand French SB0120 strains exhibit allele 4, 5, and 6 in *locus* 2461, while the Swiss *M*. *bovis* and the French cat from 1989 show a 7. Omitting marker 2461, two other clustering genotypes belonging to the widespread family of Alpine strains match the seven *loci* tested in France. Some of these strains were isolated from regions very close to the origin of the Swiss outbreak, such as Haute-Savoie, Savoie and Isère. It could therefore be suggested that since the end of the last century, a new profile showing a SLV in *locus* VNTR 2059, has been circulating in the bordering region between France and Switzerland, causing a recent, major outbreak in western Switzerland (Maria Laura Boschiroli, personal communication).

Mycobacteria from five MTBC RT-PCR positive samples could not be isolated in culture. The role played by these animals and their significance in the transmission of the pathogen may have an important impact on further bTB control. In fact, three out of five RT-PCR positive and culture negative samples originated from contact farms where no other positive animals were detected, suggesting a recent transmission of the pathogen and a low mycobacterial load of the specimens. The minimal mycobacterial load in analyzed tissue may implicate inconsistency between different detection techniques. This could be attributed to either the limit of detection of the adopted method, the distribution of mycobacteria in the tissue or their viability. An additional explanation for the divergence between RT-PCR and culture analysis could be the specificity of *artus M*. *tuberculosis* PCR (85.1%) which may lead to false positive results compared to the gold standard [36]. In the authors’ opinions the detected animals may represent eventual pathogen spreaders. Similarly to tuberculin reactors without visible lesions and that may produce a negative result in culture, these animals should be eliminated in case of contact tracing with confirmed bTB cases.

Analogous to *M*. *bovis* SB0120, *M*. *caprae* SB0418 has been reported in several continental countries [35, 37]. The analyzed Swiss isolates shared an identical discriminatory MIRU-VNTR profile with an endemic Austrian strain known as “Lechtal” type [10]. In Switzerland, the tradition of pasture on pre-alpine and alpine regions during the summer months remains very common. Herds from different farms often share the same fields and Swiss livestock can commonly be observed grazing in bordering countries where bTB has been documented both in cattle and in wildlife [10]. Since the beginning of the current century, cattle from the west Austrian regions of Vorarlberg and Tyrol, bordering the Swiss Cantons of St. Gallen and Grisons, have shown gross lesions caused by bTB at meat inspection. During this period, the Austrian Agency for Health and Food Safety (AGES) has also reported sporadic cases of tuberculosis in free-ranging red deer within the same provinces. Considering that direct contact is not necessary for the transmission of the pathogen, grazing cattle may be exposed to high risk of infection when pasturing fields contaminated by excretions such as saliva, feces, urine or discharged pus. In autumn, Swiss herds return to their farms of origin and it is not uncommon that single animals are sold to other holdings. Cattle movements such as these can have problematic results when bTB outbreaks are revealed, making nationwide monitoring programs fundamental. A clear correlation between the summer pasturing region Vorarlberg and two out of four *M*. *caprae* affected farms has thus been confirmed. Epidemiological tracing based on the Swiss national stock movement database elucidate a direct link between livestock of these two farms and the remaining infected cattle. The source of infection remains uncertain, however, as it could be either infected Austrian domestic ruminants or wild animals. Contrary to prior assumptions, a recent study performed under natural weather conditions demonstrated the long persistence of infectious *M*. *bovis* in the environment, such as water and hay (up to 58 days), strongly suggesting that indirect transmission could play an important role in bTB infection chains [38]. For this reason wildlife reservoirs are now considered to play a crucial role in eradication programs of bTB and represent an important challenge in the elimination of this zoonosis. An ongoing surveillance project started by the Swiss Federal Food Safety and Veterinary Office (FSVO) will propose an outlook about the prevalence of bTB pathogens in wild ungulates residing in the Eastern part of Switzerland.

Interestingly, one of the *M*. *caprae* SB0418 analyzed isolates, No. 13–450, showed a SLV. This variability resulted from the loss of two repeat units in addition to numerous single-base mutations randomly distributed throughout the flanking region of *locus* MIRU 40 (S3 File). This phenomenon has been reported by Roring *et al*. [27] in *M*. *bovis* field isolates from Northern Ireland and the Republic of Ireland, where heterogeneity in the nucleotide sequence of different isolates has been described. To the authors’ knowledge such genomic variations have never been disclosed before in the *M*. *caprae* species and further research is needed to identify and understand the involved mechanisms. To conclude, this study represents the first MLVA and spoligotyping analysis of Swiss bTB mycobacterial isolates. The obtained results demonstrate that the QIAxcel Advanced System as well as the ArrayMate platform provide efficient support to MLVA and spoligotyping for MTBC members causing bTB and therefore have great potential to become useful tools for research and diagnostics in veterinary laboratories. Since a panel of 49 markers represents a time- and cost-intensive effort that could not be performed for routinely intended analysis, further, more substantial investigations are needed to establish a discriminatory set of 10–15 markers specifically indicated for bTB agents, both at regional and international level.

## Supporting information

S1 Dataset PCR primer sequences and characteristics of the 31 additional MIRU-VNTR analyzed loci. (XLSX)

S1 File Expected allele calling table of the six new proposed VNTR markers. (TIF)

S2 File Annealing positions and plot of the six new proposed VNTR markers shown on reference strain H37Rv sequence. (TIF)

S3 File Comparison of the sequenced *M*. *caprae* 13–450, long fragment (A) and short fragment (B), to *M*. *tuberculosis* H37Rv in locus MIRU 40. *M*. *caprae* 13–450 long fragment shows two 54 bp long repeat units, labeled in green (A). In *M*. *caprae* 13–450 short fragment the two repeat units and 9 nucleotides of its flanking sequence are absent. Furthermore, numerous single-base mutations (31) could be distinguished in red (B). *M*. *tuberculosis* H37Rv exhibit one repeat unit in MIRU 40. Forward and reverse primers are annotated in purple.
(PDF)
