# Supplementary figures and images for: Epidemiological tracing of bovine tuberculosis in Switzerland, multilocus variable number of tandem repeat analysis of Mycobacterium bovis and Mycobacterium caprae
Source: PLoS One. 2017 Feb 21;12(2):e0172474. doi: 10.1371/journal.pone.0172474 (PMC5319696; doi:10.1371/journal.pone.0172474)

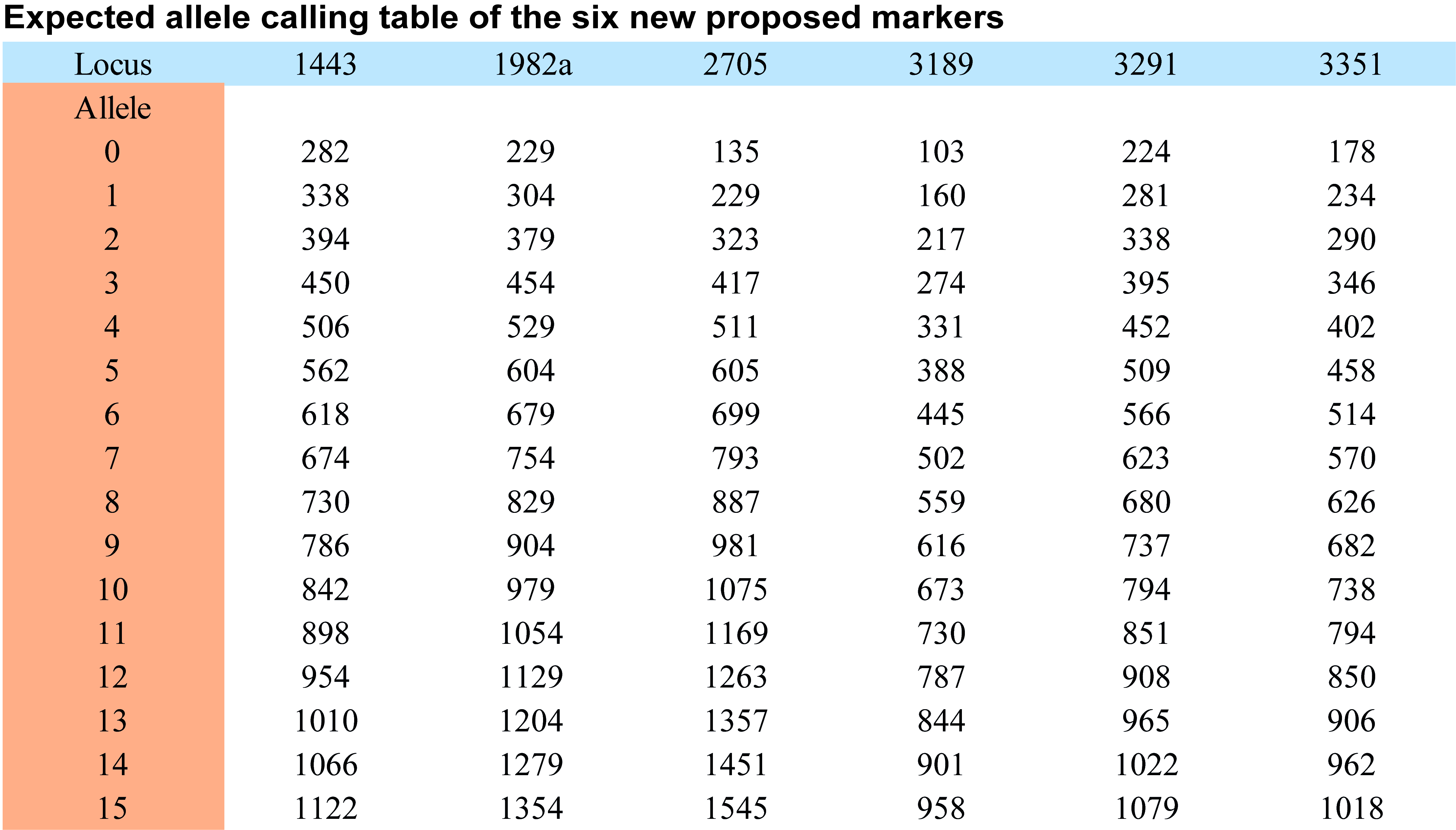

Supplement: S1 File — (TIF) [file pone.0172474.s002.tif]
