# Supplementary material for: Epidemiological tracing of bovine tuberculosis in Switzerland, multilocus variable number of tandem repeat analysis of Mycobacterium bovis and Mycobacterium caprae
Source: PLoS One. 2017 Feb 21;12(2):e0172474. doi: 10.1371/journal.pone.0172474 (PMC5319696; doi:10.1371/journal.pone.0172474)
Supplement: S3 File — M. caprae 13–450 long fragment shows two 54 bp long repeat units, labeled in green (A). In M. caprae 13–450 short fragment the two repeat units and 9 nucleotides of its flanking sequence are absent. Furthermore, numerous single-base mutations (31) could be distinguished in red (B). M. tuberculosis H37Rv exhibit one repeat unit in MIRU 40. Forward and reverse primers are annotated in purple. (PDF) [file pone.0172474.s004.pdf]

A

Forward Primer

Strain: H37Rv GGGTTGCTGG ATGACAACGT GTACACGTTT TTGGTGCGCC CGGATTCCAA CAAGACGCAG ATCAAGATCG CCGTCGAGAA GATTTTTGCC GTCAAGGTCG 100

Strain: 13-450 long GGGTTGCTGG ATGACAACGT GTACACGTTT TTGGTGCGCC CGGATTCCAA CAAGACGCAG ATCAAGATCG CCGTCGAGAA GATTTTTGCC GTCAAGGTCG 100

Strain: H37Rv CATCGGTGAA CACCGCGAAC CGGCAGGGCA AGCGTAAACG CACCCGGACC GGATACGGCA AGCGCAAGAG CACCAAGCGC GCCATCGTCA CCCTGGCGCC 200

Strain: 13-450 long CATCGGTGAA CACCGCGAAC CGGCAGGGCA AGCGTAAACG CACCCGGACC GGATACGGCA AGCGCAAGAG CACCAAGCGC GCCATCGTCA CCCTGGCGCC 200

Strain: H37Rv GGGCAGCAGG CCGATCGACC TGTTCGGGGC ACCGGCCTAG CCCGGCGACG ATGCAGAGCG AAGCGATGAG GAGGAGCAGG GCAATGCGGC CTAGCCCGGC 300

Repeat Unit 1

Strain: 13-450 long GGGCAGCAGG CCGATCGACC TGTTCGGGGC ACCGGCCTAG CCCGGCGACG ATGCAGAGCG AAGCGATGAG GAGGAGCAGG GCAATGCGGC CTAGCCCGGC 300

Strain: H37Rv ---------- ---------- ---------- ---------- ---------- ----GACGAG AGCGTGAGAG AAAGACCTGA TTAGACATGG CAATTCGCAA 346

Repeat Unit 2

Strain: 13-450 long GACGATGCAG AGCGAAGCGA TGAGGAGGAG CAGGGCAATG CGGCCTAGCC CGGCGACGAG AGCGTGAGAG AAAGACCTGA TTAGACATGG CAATTCGCAA 400

Reverse Primer

Strain: H37Rv GTACAAGCCC ACGACGCCTG GTCGTCGCGG CGCCAGCGTA TCTGATTTCG CCGAGATCAC CC 408

Strain: 13-450 long GTACAAGCCC ACGACGCCTG GTCGTCGCGG CGCCAGCGTA TCTGATTTCG CCGAGATCAC CC 462

B

Forward Primer

Strain: H37Rv GGGTTGCTGG ATGACAACGT GTACACGTTT TTGGTGCGCC CGGATTCCAA CAAGACGCAG ATCAAGATCG CCGTCGAGAA GATTTTTGCC GTCAAGGTCG 100

Strain: 13-450 short GGGTTGCTGG ATGACAACGT GTACACGTTC GTCGTGCACC CGGACTCGAA CAAGACGCAG ATCAAGATCG CGGTGGAGAA GATCTTCTCG GTCAAGGTCG 100

Strain: H37Rv CATCGGTGAA CACCGCGAAC CGGCAGGGCA AGCGTAAACG CACCCGGACC GGATACGGCA AGCGCAAGAG CACCAAGCGC GCCATCGTCA CCCTGGCGCC 200

Strain: 13-450 short CGTCGGTGAA CACCGCCAAT CGGCAGGGCA AGCGCAAGCG CACCAGGACC GGATTCGGCA GGCGCAAGAG CACCAAACGC GCCATCGTCA CCCTGGCCCC 200

Repeat Unit 1

Strain: H37Rv GGGCAGCAGG CCGATCGACC TGTTCGGGGC ACCGGCCTAG CCCGGCGACG ATGCAGAGCG AAGCGATGAG GAGGAGCAGG GCAATGCGGC CTAGCCCGGC 300

Strain: 13-450 short GGGCAGCAAG CCGATCGACC TGTTCGGAGC GCCGGCCTAG CC-------- ---------- ---------- ---------- ---------- ---------- 242

Reverse Primer

Strain: H37Rv GACGAGAGCG TGAGAGAAAG ACCTGATTAG ACATGGCAAT TCGCAAGTAC AAGCCCACGA CGCCTGGTCG TCGCGGCGCC AGCGTATCTG ATTTCGCCGA 400

Strain: 13-450 short -----GCGCG CGAGAGAGAG ACCTGATAAG ACATGGCAAT TCGCAAGTAC AAGCCGACGA CACCGGGTCG TCGCGGCGCC AGCGTATCTG ATTTCGCCGA 337

Strain: H37Rv GATCACCC 408

Strain: 13-450 short GATCACCC 345
